# Supplementary material for: Acoustic radiation-free surface phononic crystal resonator for in-liquid low-noise gravimetric detection
Source: Microsyst Nanoeng. 2021 Jan 18;7:8. doi: 10.1038/s41378-020-00236-9 (PMC7812345; doi:10.1038/s41378-020-00236-9)
Supplement: Supplementary file 1 — Supplemental material [file 41378_2020_236_MOESM1_ESM.docx]

Supplementary Information

**Acoustic-radiation free phononic biosensor for in-liquid low-noise gravimetric detection**

Feng Gao^1*^, Amine Bermak^1^, Sarah Benchabane^2^, Laurent Robert^2^, and Abdelkrim Khelif^2^

^1^ College of Science and Engineering, Hamad Bin Khalifa University, Education City, Doha, Qatar

^2^ Institut FEMTO-ST, CNRS, Université de Bourgogne-Franche-Comté, Besançon, France

* Corresponding author: Feng Gao (fgao@hbku.edu.qa)


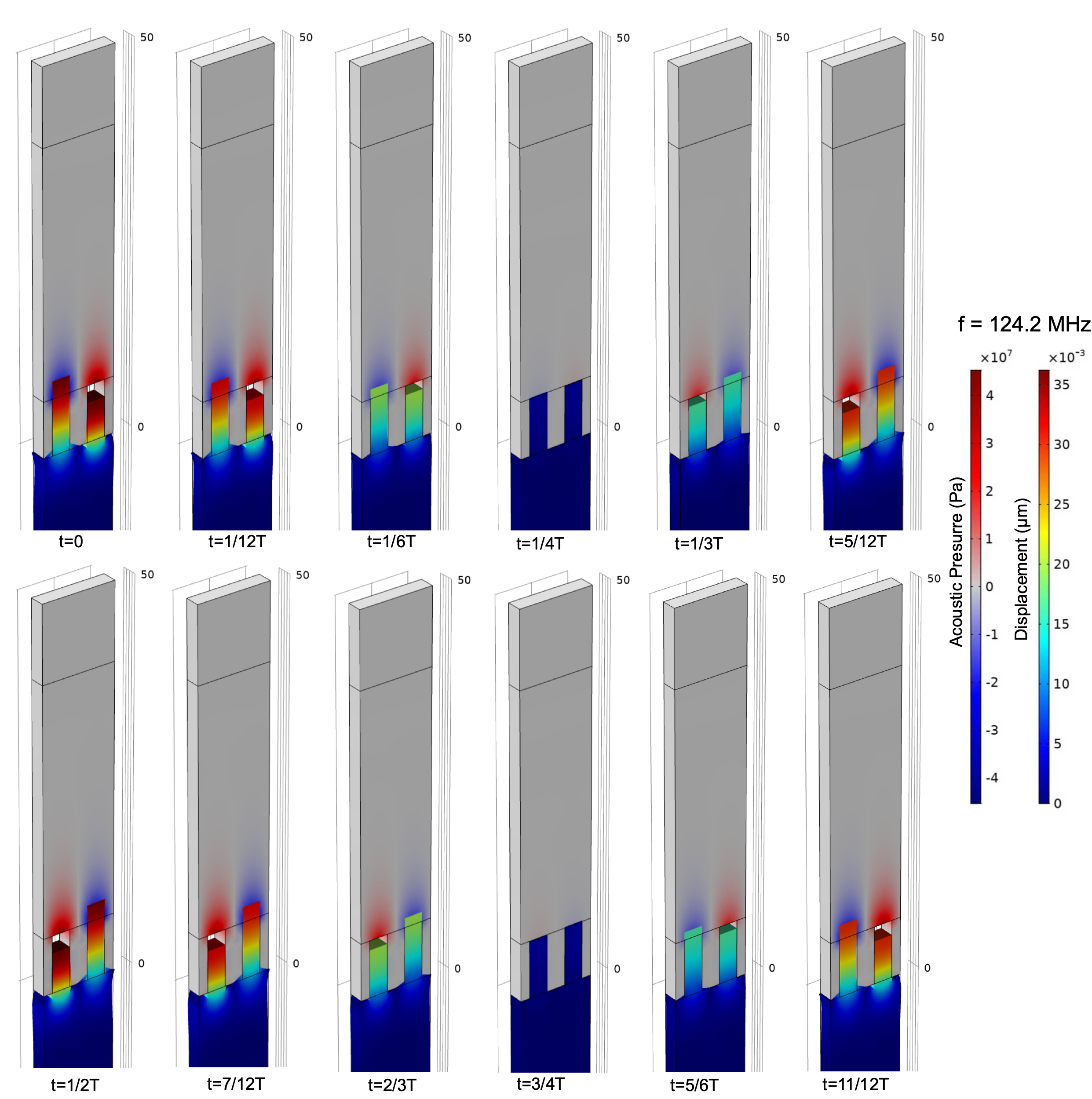


*Figure S1. The substrate displacement and acoustic pressure distribution of the SPC resonator at different time nodes in a full oscillation period (T).*

*Figure S2. The unit cell of the SPC resonator used in FEM analysis. Model simplification is introduced by the periodic boundary conditions and PMLs to accelerate the numerical simulation.*

*Figure S3. FEM modelling unit cell of the (a) positive photoresist (pPR) profile SPC resonator, (b) negative photoresist (nPR) profile SPC resonator and (c) conventional SH-SAW resonator. A polymer layer (PMMA) is used as the mass loading layer. The frequency shift in response to the PMMA density variation is used to obtain the mass sensitivity under homogeneous full coverage condition.*


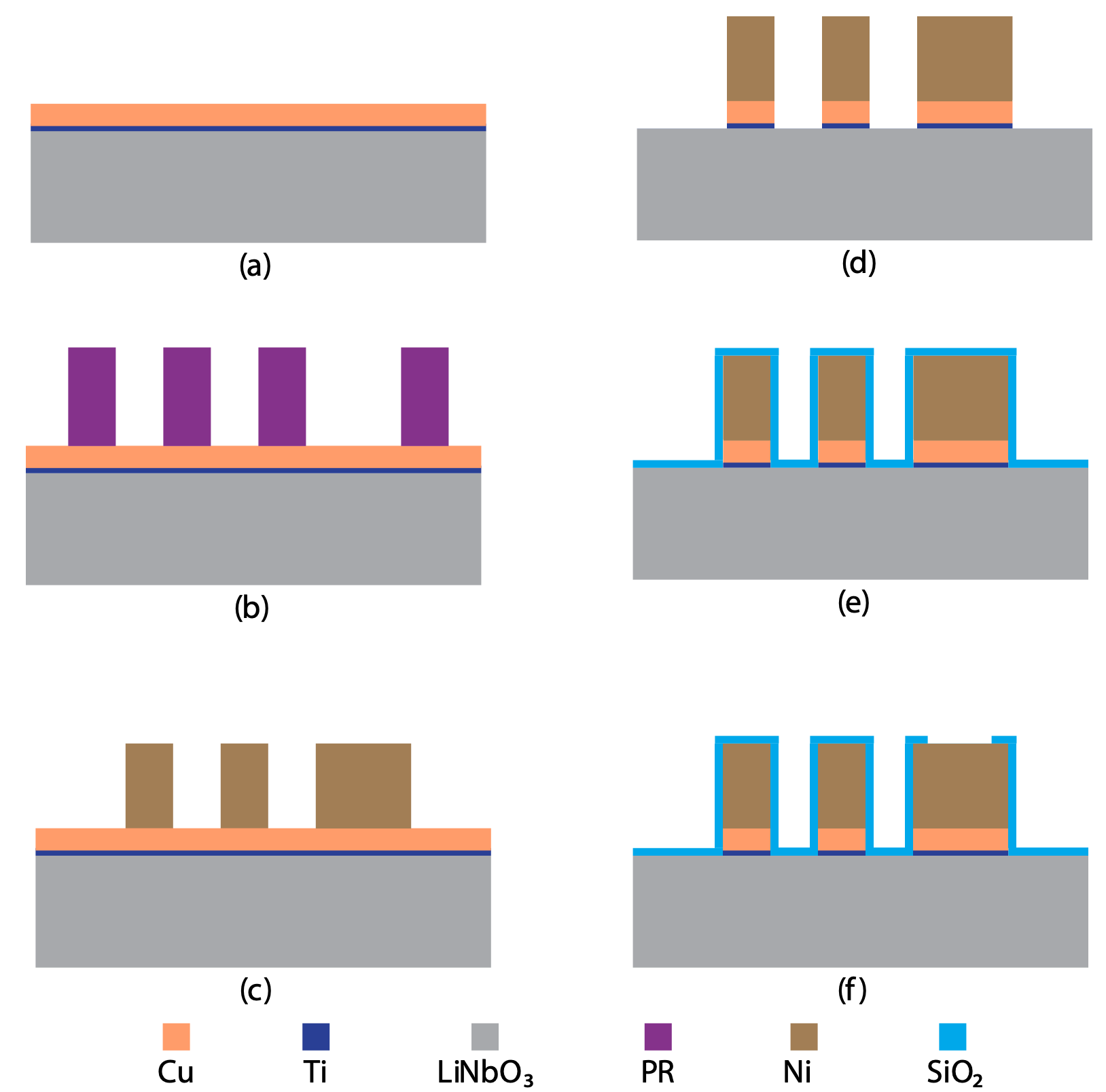


*Figure S4. Fabrication process flow of the SPC biosensor.*

Step (a): Deposit 20 nm titanium (Ti) and 100 nm copper (Cu) on the 128° Y cut lithium niobate (LiNbO_3_) substrate. The copper layer is the seed layer for subsequent nickel (Ni) electroplating. The titanium layer is used to promote the adhesion between the lithium niobate substrate and copper layer.

Step (b): Produce the electroplating mold by photolithography with thick photoresists (PR). Two photoresists including a positive photoresist (AZ9260) and a negative photoresist (AZnLOF2070) are used in this step to produce PR molds with trapezoid and inverted trapezoid electrode profiles respectively. The thicknesses of both photoresists are 8 $\mu m$.

Step (c): Electroplate the high aspect ratio nickel electrodes to form the phononic crystals. After the electroplating, the photoresist mold is removed by immersing the wafer in Microposit 1165 remover at 70 $℃$ overnight.

Step (d): Remove the copper and titanium seed layer by reactive ion etching (RIE). This step separate the metal electrodes electrically to form the IDT.

Step (e): Deposit 200 nm silicon dioxide (SiO_2_) passivation layer by chemical vapor evaporation.

Step (f): Remove the SiO_2_ layer covering the PADs for bonding wire connection. A thin photoresist (AZnLOF 2020) was used as the etching mask, while RIE is used for the SiO­_2_ removal.
